# Highlights from Choosing Wisely 2022 for Resource Limited Settings: Reducing Low Value Cancer Care for Sustainability conference, 17th-18th September, Mumbai, India

**DOI:** 10.3332/ecancer.2022.1465

**Published:** 2022-11-03

**Authors:** Amol Akhade, Bishal Gyawali, Richard Sullivan, Bhawna Sirohi

**Affiliations:** 1 BYL Nair Hospital and TN Medical College, Mumbai 400008, Maharashtra, India; 2 Queen’s University, Kingston, ON K7L 3N6, Canada; 3 King’s College London, London WC2R 2LS, UK; 4 Balco Medical centre, Raipur 493661, Chhattisgarh, India

**Keywords:** choosing wisely, high value interventions, cost effectiveness

## Abstract

The ‘Choosing Wisely 2022’ conference, organised by the ecancer foundation, was held at the Tata Memorial Hospital, Mumbai, India, on 17 and 18 September. It was a successful event with 159 delegates attending it in person and around 328 delegates attending online. Thirty oncology experts from across the world shared their thoughts during this meeting. The theme of the conference was to focus on cancer care, in low- and middle-income countries (LMICs). The emphasis of discussion was on ways to select more cost-effective and high value treatments and interventions and minimise financial toxicity. In addition, cancer research from LMICs needs to be improved substantially. Collaboration and networking amongst cancer institutions in LMICs is essential.

## Introduction

Cancer treatment is evolving rapidly. Newer drugs, especially immunotherapy and targeted agents, are being approved at a rapid rate, oftentimes before comprehensive evidence of efficacy and safety are established. While some of these interventions have improved outcomes for patients, studies have shown that a majority of these new cancer drugs and high-end technologies are of only marginal benefit and are not accessible in most low- and middle-income countries (LMICs). Global inequalities in cancer care are increasing and the gap between LMICs and high-income countries (HICs) is widening [1]. Political turmoil, war and global economic recession are further widening cancer treatment inequalities. LMICs also spend heavily on low-value interventions while lacking the funds for high-value interventions. Hence, it has become very important to segregate and raise awareness of low-value treatment options from high-value, cost-effective treatment options, in cancer care.

With this aim, the Choosing Wisely conference was organised by the ***e*cancer foundation at the Tata Memorial Hospital (TMH), Mumbai,** India, on 17 and 18 September 2022. **Balco Medical centre,** Raipur, Chhattisgarh and the **National Cancer Grid, India** were joint organisers. It was a hybrid meeting allowing for both in-person and online participation. The opportunity to meet and interact with experts from various fields of oncology, both from India and abroad, was very exciting for the participants. Indeed, the conference was enthusiastically attended by 159 delegates in person and 328 delegates on line. The emphasis of the event was to share thoughts on how to improve access to high-value care, avoid low-value care and minimise financial toxicity. The conference was preceded by a workshop on critical aspects of clinical research especially for LMICs.

## Highlights of day 1

The pre-conference workshop on day 1 included a series of lectures by various international and national experts on different aspects of clinical trials and research. **Dr Scott Berry**, from Queen’s University, Kingston, Canada, highlighted the importance of clinical research in improving patient outcomes. He stated that by 2030, approximately 75% of cancer deaths will occur in LMICs [2] and hence, cancer research in LMICs should be a priority. Currently, only 8% of clinical trials come from LMICs. In fact, many countries in sub-Saharan Africa have no ongoing clinical trials [3]. To improve this situation, LMIC governments must allocate more funds to clinical research and promote the culture of research at various levels. We need to develop cooperative research networks such as the National Cancer Grid in India.

**Dr Ian Tannock**, from Princess Margaret Cancer Centre and University of Toronto, Canada, talked about developing low-cost strategies for existing therapies. He suggested strategies such as using lower dose or less frequent dosing (immunotherapy drugs), shorter duration of treatment (6 months of adjuvant trastuzumab instead of 1 year), dose modifiers including food (abiraterone) and therapeutic substitution (use of generics, wherever approved) [4]. Dr Tannock applauded the conduct of low-dose immunotherapy trials in India (the use of low-dose, 20 mg nivolumab for palliation of head and neck cancer in TMH by Dr Vijay Patil has shown significant improvement in overall survival compared to chemotherapy) [5].

**Dr Chris Booth**, from Queen’s University, Kingston, Canada, discussed the increasing use of surrogate endpoints in oncology trials. He emphasised that only two endpoints really matter to patients – increase in overall survival and improvement in quality of life [6, 7]. He stressed the fact that progression free survival is not a valid surrogate for overall survival (OS) or quality of life. He also pointed out publication bias in leading oncology journals against authors from LMICs [1]. He showed evidence that cancers of ‘poverty’, i.e., those cancers occurring in LMICs, are underrepresented in clinical trials globally (e.g. cervix, head & neck cancer) [8, 9]. There is a real crisis of ‘value’ in cancer care: high drug prices, low-value interventions and questionable endpoints are responsible for this crisis.

**Dr Bishal Gyawali**, from Queen’s University, Kingston, Canada, spoke about how to choose an appropriate control arm representing the current standard of care in clinical trials. He explained how some trials choose inferior control arms by either using an outdated control arm, or not allowing an effective drug in the control arm, or offering a placebo instead of active treatment. He also indicated how inferior post-progression treatment given to patients recruited to control arms results in exaggerated OS benefit for the experimental agent. He described how low-value trials with modest beneficial results are given positive spin in publications. Another concern is inappropriate cross-over in clinical trials which can lead to exaggerated results [10, 11]. He suggested that locally developed generic, ‘me-too’ drugs can help to reduce drug costs. LMICs should focus on clinical trials tackling locally prevalent cancers with local generic drugs [12].

**Dr Kumar Prabhash**, from TMH, Mumbai, India, also emphasised that LMICs need to focus on local problems and undertake locally useful clinical trials. **Dr Pramesh**, also from TMH, Mumbai, India, discussed the development of networks in LMICs for initiating and executing clinical trials and stimulating multicentric collaboration. He suggested that unlike HICs, the culture of collaboration is lacking in LMICs and needs to be nurtured so as to have improvements in trial standards. **Dr Sudeep Gupta**, from TMH, Mumbai, India, discussed the statistical analysis of clinical trials. He explained the concept of hazard ratio and how to interpret Kaplan–Meier survival curves. An important concept is magnitude of benefit. A modest hazard ratio in a population with poor outcome in the control arm will lead to a large absolute clinical benefit, but a strong hazard ratio with excellent outcome in the control arm will still lead to a smaller absolute clinical benefit. It is necessary to interpret the hazard ratio carefully when interpreting usefulness of trial results in clinical practice. The session ended with **Dr Vanita Noronha**, from TMH, Mumbai, India, describing the difficulty faced by researchers in LMICs. Lack of funds for investigator-initiated trials, long times for ethics approvals, lack of sufficient human resources for running the trials, overall lack of enthusiasm amongst LMIC oncologists for doing research due to excessive clinical work and lack of incentives for doing research were the main points discussed in a lively panel discussion.

The afternoon session began with the inaugural **Gordon McVie Lecture** by **Dr RA Badwe**, from TMH, Mumbai, India, on how to have sustainability in delivering quality health care in low-resource settings. He showed that simple timely interventions can lead to large benefits. He emphasised collaboration, team efforts and focusing on local problems at a grass-roots level, so as to develop long-term sustainable and effective cancer care. **Dr Bhawna Sirohi**, from Balco Medical centre, Raipur, Chhattisgarh, emphasised that the need for choosing wisely in low-resource settings is an ethical imperative. **Dr Pramesh** also discussed the need for choosing wisely in low-resource settings and described efforts taken by the National Cancer Grid in India. **Dr Bishal Gyawali** spoke about how to define value-based care for LMICs. He explained that value depends upon both cost and efficacy. Cost control is a complex issue with multiple factors but choosing interventions with better magnitude of benefit is in the clinicians’ hands. Clinicians, policy makers, regulators and all stakeholders, including patients, need to be aware about the real value of a drug or any other intervention.

**Dr Richard Sullivan**, from King’s College London, UK, discussed the impact of the current war in Ukraine on its cancer care. Economic recession is further affecting cancer care which was already impaired due to the Covid pandemic. These factors are increasing the gap between HICs and LMICs and we need to make conscious and persistent efforts to reduce this gap. The newer technologies and the pharmaceutical industry are driving the economics of cancer care. He suggested that we need to look beyond technology for improvement in cancer care as it will probably increase the cost of the care without any meaningful improvement in end results. **Dr Susie Stanway**, from the UK Global Cancer Network**,** took this discussion further and explained the importance of collaborative networks and the role they play in improving cancer care. The collaborative networks in LMICs and HICs need to join hands to improve global cancer care.

The last session of day 1 was about the top ten choosing wisely interventions for common cancers, discussed by different oncologists. **Dr Amol Akhade**, from Nair Hospital, Mumbai, stressed avoidance of Hyperthermic Intraperitoneal Chemotherapy (HIPEC) in GI cancers, as it has no evidence of benefit and can even be detrimental; it also leads to financial toxicity [13, 14]. He also recommended against the use of circulating tumour cells outside the setting of a clinical trial in LMICs as we are not sure about the clinical meaningfulness of such tests. **Dr Amandeep Arora**, from TMH, Mumbai, India, talked about avoiding robotic surgery for urinary bladder cancer as its added advantage over laparoscopic surgery is minimal and not cost-effective. He also recommended observation alone for men with low-grade prostate cancers if asymptomatic. **Dr Kumar Prabhash** emphasised avoiding robotic surgery for intra-oral cancers, and encouraged enrolling patients on low-dose immunotherapy trials for advanced head neck cancers. He also stated that there is no benefit of cetuximab over cisplatin for concurrent chemoradiation in platinum eligible patients. For patients not eligible for cisplatin, he suggested 20 mg docetaxel as a useful and cost-effective option [15].

## Highlights of day 2

**Dr Ian Tannock** spoke about defining clinically meaningful outcomes for clinical trials, including quality-of-life endpoints. Very few cancer trials include quality of life or patient-reported outcomes as a major end point and most of them don’t measure it properly [16]. He highlighted the need to include and publish patient-reported outcomes rigorously. **Dr Christopher Booth** spoke about Time Toxicity, i.e., the time taken out of patients’ lives while in hospital getting cancer care. Especially when time is limited due to incurable disease in the advanced settings, patients will like to spend less time in hospital and more time at home, off treatment. Randomised control trials need to be done to evaluate whether treatment improves home days as one of the end points. He stressed that with information on time toxicity, a clinician can better guide a patient to align treatments with patient goals, thereby improving their quality of life [17].

**Dr Sudeep Gupta** conducted a lively panel discussion of the challenges in raising the bar in clinical trials in India. **Dr Vijay Patil**, from TMH, Mumbai, India, stressed that the most important factors required to conduct successful clinical trials in LMICs were time, motivation and collaboration. The panel agreed that the control arm in LMIC trials should be as per the local standard of care, as that is what is cost-effective and practical. **Dr Rajagopal**, from the Pallium India Foundation, India, stressed the importance of palliative care in cancer care. Palliative care needs to be integrated early in the disease course, rather than as the last option. Appropriate use of analgesia with drugs like opiates should be initiated early. We should avoid unnecessary ICU admissions for terminally ill cancer patients. We need to focus on nutritional aspects early in cancer care, especially in LMICs, as many of our patients are malnourished**. Dr Aju Mathews**, from Ernakulam Medical Centre, Kerala, India, led a panel discussion where the importance of patient education to improve cancer care was discussed in detail. Panellists agreed that patients need to be at the centre of treatment decisions and the main focus should be on patient-reported outcomes. The meeting ended with an important meeting of **patient advocacy groups** in which ways to improve coping mechanisms for patients and their families and how to help them during the journey of cancer treatment were discussed.

## Conclusion

The Choosing Wisely 2022 for resource limited settings: reducing low value cancer care for sustainability meeting was well attended by oncologists, both in-person and virtually. LMICs need to focus on clinical trials looking at locally prevalent cancers with appropriate control arms. Randomised controlled trials (RCTs) relevant to the local settings should be a priority over participating in international RCTs with unethical control arms done solely to gain drug approval elsewhere. Investigators in LMICs should engage in multicentric collaboration so that together they can overcome hurdles faced in research. Persistence, motivation and working together are key to having more high-quality impactful research from LMICs. Low-value surgical interventions, technologies and drugs should be avoided by oncologists, both from LMICs and HICs. The onus on delivering affordable quality cancer care, in resource-constrained settings, lies on all stakeholders including government, oncologists, pharmaceutical companies and patient advocacy groups. Together we can make a difference if we choose wisely.

## Conflicts of interest

None.

## Funding

None.
